# Can *Mimosa pudica* Plants Enumerate Light Exposure Events?

**DOI:** 10.1111/cogs.70161

**Published:** 2025-12-28

**Authors:** Peter M. Vishton, Paige J. Bartosh

**Affiliations:** ^1^ Department of Psychological Sciences, William & Mary

**Keywords:** Plant learning, Plant cognition, Enumeration, Extra‐neuronal learning

## Abstract

Plants sense and respond to information present in their surrounding environment. Recent work has sought to characterize the limits of these information processing abilities. Here, we present evidence that the movements of *Mimosa pudica* plants are mediated by the number of illumination events to which they have been exposed. The plants were repeatedly presented with 2 days in which light was provided for half of the day, followed by a third day in which light was not provided. The nyctinastic movements of the plants shifted to follow this light–light–dark pattern. During early, dark hours prior to light onset, the plants moved more on days in which light was likely to be provided and less on days in which light was unlikely. This movement tendency was not present during the initial weeks of the study. The plants altered their movement patterns over 15 days in a fashion that is well fit by a logarithmic function. To test whether plant movement was based on temporal factors, rather than event enumeration, we altered the lengths of day–night cycles in the second and third phases of the study. After accommodating their motion to follow a 3 × 24‐h light–light–dark cycle, the plants immediately generalized their performance after an abrupt shift to a 3 × 20‐h cycle. In Study Phase 3, the day length was randomly varied between 10 and 32 h after every light–light–dark cycle. The plants exhibited key movement patterns when randomly selected day durations were between 12 and 24 h. Although higher levels of variability were apparent, the movement levels of the plants seemed to be modulated by the number of light exposure events. The results provide evidence that plants, and perhaps other non‐neuronal tissues, may be capable of processing enumeration‐related information, although replication with additional controls is needed.

## 1 Introduction

Nearly all living organisms actively gather, encode, and utilize information from their environments to support adaptive biological functions. A central aim of cognitive science is to characterize the systems by which organisms acquire, store, and process such information to guide behavior and decision‐making (Anderson, 2013; Gallistel, 1990). While the majority of research in this area has focused on animals, emerging studies suggest that similar computational processes may be observed in plants (Calvo & Friston, 2017; Trewavas, 2003).

Plants lack neurons, a fact that has historically contributed to the assumption that they are incapable of cognitive‐like processes such as memory, learning, and inference. However, mounting evidence challenges this view by demonstrating that plants can exhibit forms of plasticity, anticipation, and decision‐making that meet behavioral criteria typically used to identify cognition in nonverbal animals (Calvo & Lawrence, 2022; Gagliano, Renton, Depczynski, & Mancuso, 2014; Thellier & Lüttge, 2013). If such processes can be implemented in the absence of neuronal tissue, this would suggest that cognition is not necessarily tied to neural architecture but may instead arise from more general biological substrates, including complex biochemical and metabolic networks.

Trewavas (2003, 2005) has argued that plants possess a form of intelligence rooted in their highly dynamic and responsive metabolic systems. According to this view, the flexible, adaptive computations usually attributed to neuronal circuits may also emerge from non‐neural cellular processes such as signal transduction, calcium oscillations, hormonal regulation, and gene expression. If so, plants could serve as powerful model systems for exploring the implementation of cognitive functions in distributed, non‐neuronal architectures.

In the present study, we examine the possibility that *Mimosa pudica* encodes and utilizes enumeration information—that is, information about discrete quantities of repeated stimuli. Enumeration requires the abstraction of numerical information from sequences of perceptual events that vary in count but not necessarily in qualitative content. In the following section, we review research supporting the claim that many nonverbal organisms can register and respond to numerosity, thereby establishing a comparative framework for evaluating numerical processing in plants.

## 2 Animals and plants encode and respond to quantity information

Many organisms gather information from their environment and use it to enhance biological function. Patterns present in auditory, visual, tactile, and other sensory domains can dictate actions that will increase the ability of organisms to survive, thrive, and reproduce. In addition to sensing the presence of some stimulus in the surrounding environment, organisms also sense the quantity of that stimulus. For instance, a foraging animal may see two visual patterns indicating the presence of food in different locations. Encoding the quantity of food present in each location will indicate where the animal should move to maximize caloric intake.

Experiments conducted with many species demonstrate that animals possess the ability to compare quantities in this fashion, using that comparison to guide foraging, social decisions, and problem‐solving. Agrillo, Miletto Petrazzini, and Bisazza (2017) reviewed studies in which various fish species were presented with two groups of conspecifics behind transparent barriers. The fish preferentially moved toward the larger group, especially when the ratio exceeded 2:1. (The preference for a larger group is associated with increased safety from predators and mate access.) (Also see Gómez‐Laplaza & Gerlai, 2011.) Chicks have similarly shown preferences for larger groups of visual elements (Rugani, Vallortigara, & Regolin, 2015). Domestic dogs have been shown to accurately distinguish between two quantities of food, choosing the larger amount in controlled trials, especially when ratios are large (Ward & Smuts, 2007). Xu and Spelke (2000) used a looking habituation paradigm to demonstrate quantity discrimination abilities in 6‐month‐old human infants. They showed babies alternating images with 8 versus 16 dots. Infants looked longer at novel quantities when the numerical difference was large enough (2:1 ratio), suggesting they registered changes in number.

Brannon and Terrace (1998) trained rhesus monkeys to touch 2D stimuli with increasing numbers of elements (1 to 4) in order. Monkeys generalized this ordering to novel sets (5 to 9), implying that they understood the concept of cardinality. Scarf, Hayne, and Colombo (2011) reported that pigeons perform similarly on the tasks used by Brannon and Terrace.

There is evidence that plants also respond to quantity information. Research has shown that plants are influenced by the number of neighboring individuals, track the quantity of pollinator visits, and regulate internal resource allocation in a way that reflects awareness of relative quantity. Several studies have demonstrated that the number of nearby plants influences root growth. Gersani, Brown, O'Brien, Maina, and Abramsky (2001) found that when individual pea plants were grown with neighbors, their root systems became more extensive than when they were grown alone, despite identical nutrient conditions. Subsequent studies examined whether plants could not only detect the presence of neighbors but also quantify them. Novoplansky (2009) proposed that plants might respond to the density of competitors, adjusting root growth in proportion to neighbor number. Supporting this, Cahill et al. (2010) demonstrated that velvetleaf plant root foraging behaviors change as a function of the number of neighbors. Specifically, individual plants allocate more root biomass to regions with fewer neighbors, suggesting a strategic avoidance of highly competitive zones.

Kessler and Baldwin (2002) describe how wild tobacco plants exposed to higher levels of simulated herbivory (via mechanical wounding) produced significantly greater concentrations of defense compounds such as nicotine and trypsin proteinase inhibitors. This suggests that plants can integrate information about the quantity of attack to modulate chemical defenses accordingly.

Some orchids and other angiosperms alter nectar production or floral presentation based on the number of pollinator visits, implying a capacity to accumulate and respond to interaction quantities. Gagliano et al. (2014) found that plants exposed to repeated environmental stimuli learn to anticipate and modify their behavior based on exposure frequency—suggesting a memory mechanism related to encounter quantity.

Trewavas (2005) argued that plant behavior, though not controlled by a nervous system, involves complex signal processing capable of interpreting relative magnitude, including quantity‐related cues. Chamovitz (2020) summarized a variety of findings suggesting that while plants lack a brain, their decentralized systems integrate environmental data in a way that resembles sensory processing—including sensitivity to changes in number of environmental interactions, such as light pulses, neighbor presence, and herbivory events. Overall, these findings suggest that plants possess a form of non‐neural quantitative assessment of their surroundings, enabling them to modulate internal function based on the quantities of many different stimuli. This capacity highlights a sophisticated level of environmental sensitivity and plasticity in plant behavior.

## 3 Do animals encode number or just number‐correlated information?: Approximate number system (ANS) versus sensory integration system (SIS)

There is an abundance of evidence that animals are sensitive to differences in stimulus quantity, but how do they encode these differences? In order to produce the behavioral patterns summarized in the prior section, there is no inherent need for a symbolic or enumerative encoding of stimuli. To pick the larger of two food stimuli, a dog could simply select the larger pile. If the food is brown, then orienting in the direction of the largest brown stimulus would result in the observed behaviors. Fish could similarly orient in the direction of the shoal stimulus that subtends the largest area of their visual field. If a plant has more potentially competitive neighbors, then there will be more roots present in the soil. The animals and plants might encode the number of competitors, but they could instead encode something that is correlated with that number. There are many potential correlations that could be used in this fashion.

Some research has sought to characterize the nature of encoding associated with these observed quantity preferences. For instance, in the Brannon and Terrace (1998) study described above, in which rhesus monkeys learned to touch 2D stimuli in order of increasing numbers of elements, the researchers varied the size and complexity of stimulus elements to decouple the correlations between number and other stimulus properties. If the Brannon and Terrace stimuli had always been blue rectangles of a particular size, presented on a green background, then the monkeys could have achieved accurate performance by touching the stimuli in order from the least blue to most blue, removing the need for any enumeration‐based encoding. These researchers varied many stimulus factors across the study. In some study conditions, as the number of stimulus elements increased, the sizes of individual elements were systematically reduced, such that the target stimuli subtended a constant amount of screen area. This, of course, creates another potential correlation. Perhaps the monkeys would choose stimulus images in order from the largest to smallest individual element size, again achieving the observed behavioral patterns without enumeration‐based encoding.

Brannon and Terrace (1998) reinforced monkeys for correct performance while varying many stimulus construction factors: element size, color, shape, and surface area. Monkeys saw individual displays repeatedly during the learning process. The authors’ strongest evidence that the monkeys were sensitive specifically to enumeration information comes from trials in which the experimenters presented novel stimuli to the monkeys—stimuli for which no reinforcement feedback had been provided yet. The monkeys generalized their learning to these stimuli in terms of the number of elements, even in situations in which other stimulus construction factors indicated a different response.

Brannon and Terrace (1998), based on Davis and Pérusse (1988), suggest that animals may engage in numerical encoding of stimuli as a “last resort,” when no simpler stimulus factors enable predictions of future events—in this case, reinforcement. This concept fits well with other studies of quantitative encoding in plants and animals. Scarf et al. (2011) used the same procedures as Brannon and Terrace to control for non‐numerical stimulus factors with pigeons and obtained similar results.

The challenge of disentangling number versus number‐correlated information arises in any study of enumeration processing with nonverbal organisms: (a) Experimenters present an organism with a structured set of stimuli that vary in terms of number. (b) Experimenters then identify some aspect of organism behavior that is a function of that enumeration. (c) Test trials must be conducted in which enumeration‐based processing predicts one response while non‐enumeration‐based processing predicts another. This approach has been used in several of the studies described here already.

This type of research has often been framed as a theoretical debate between proponents of an ANS and advocates of a SIS. The ANS hypothesis proposes that the nervous system encodes numerosity—an abstract, modality‐independent representation of set size that follows Weber's law and supports later symbolic mathematical abilities (Dehaene, 2011; Van Hoogmoed & Kroesbergen, 2018). Neural and behavioral evidence from humans and other species suggests that such representations may be supported by dedicated number‐sensitive mechanisms (Clarke & Beck, 2021). In contrast, the SIS account argues that apparent numerosity perception arises not from a distinct numerical mechanism but from the weighted integration of correlated continuous magnitudes such as total area, density, item size, and other attributes of sensory inputs (Gebuis, Kadosh, & Gevers, 2016; Gevers, Kadosh, & Gebuis, 2016). According to this view, observers infer numerosity indirectly through the convergence of visual cues rather than computing the number directly.

The ANS approach is supported by studies such as Brannon and Terrace (1998) and Scarf et al. (2011) suggesting that animals remain capable of encoding differences in stimulus number even when other correlated factors are eliminated (also see Canfield & Smith, 1996; Feigenson, Dehaene, & Spelke, 2004; Pepperberg, 2006; Rugani et al., 2015; Ward & Smuts, 2007; Wynn, 1992; Xu & Spelke, 2000).

Another example of the ANS approach comes from the work of Agrillo et al. (2017), who described studies in which fish preferentially moved toward the larger of two conspecific groups. The larger group consisted of a greater number of fish, but the larger group also exhibited a range of other correlated differences: larger visual angle, more visible motion, and greater stimulus density. By selecting larger individual fish for inclusion in the smaller groups and smaller individuals for the larger groups, the experimenters were able to hold the overall visual angle constant. By reducing the water temperature for the larger group, the amount of movement could be reduced, thus eliminating the movement differences. By placing the smaller group of fish in a smaller container, the stimulus density could be controlled. In general, when researchers have removed these correlated stimulus differences, the performance accuracy of the fish was reduced. That is, the fish failed to discriminate group size unless the ratio of large‐to‐small group members was large.

While debates on this topic have sought to identify whether the ANS or SIS perspective is correct, more recent work has suggested that both systems may contribute to performance in nonsymbolic number tasks. For instance, Abalo‐Rodríguez, De Marco, and Cutini (2022) demonstrated that numerosity judgments are jointly shaped by discrete number information and continuous visual cues, and that calibration procedures can selectively increase the weight assigned to numerical information. Such findings suggest that “number” perception reflects a dynamic interplay between an abstract numerical code and the sensory evidence from which it is inferred (Zanon, Potrich, Bortot, & Vallortigara, 2022). This integrative perspective implies that number representations are neither entirely independent of sensory input nor reducible to it—highlighting the importance of precise stimulus control and cross‐species methodological consistency in future work.

## 4 Do plants encode number or just number‐correlated information?

There is evidence that plants are capable of responding to quantity information but to our knowledge there is no evidence that plants encode this information in terms of enumeration. For instance, there is evidence that the quantity of nearby plants causes greater root growth, but more recent studies have presented evidence that the presence of below‐ground signaling molecules such as strigolactones and auxins causes this (Badri, Zolla, Bakker, Manter, & Vivanco, 2013). More nearby plants result in greater concentrations of these molecules, which in turn stimulate root growth. The plants clearly encode the signaling molecule concentration, but there is no evidence that the number of neighboring plants is encoded.

Similarly, with wild tobacco, while there is evidence that being attacked by more predators results in a greater production of nicotine, it is the correlated magnitude of the leaf damage that is key. As leaf cells are damaged, a cascade of chemical and transcriptional events takes place to increase nicotine production (Kessler & Baldwin, 2002). For instance, the plants would not distinguish between one predator versus two predators that eat leaves half as fast.

Overall, research on plant information processing does not support the claim that plants encode enumeration information as opposed to enumeration‐correlated quantity information. The study we present here explores the hypothesis that plants do encode enumeration information.

## 5 Adaptive value and computational possibility of enumeration encoding for plants

Before exploring evidence that plants are capable of encoding and responding to enumeration information, it is important to consider why such a capacity would hold adaptive value for nonverbal organisms—whether animal or plant. Several researchers have argued that the ability to process enumeration information plays a fundamental role in enabling organisms to make context‐sensitive, adaptive decisions that enhance survival and reproductive success (Dehaene, 2011; Gallistel, 1990). Both Randy Gallistel and Stanislas Dehaene have proposed that numerical cognition is a core cognitive function, deeply embedded in biological systems due to its broad utility across domains such as foraging, navigation, social coordination, and risk evaluation.

Gallistel emphasized that numerical representations serve as an abstract, amodal format for encoding variables like time, space, and quantity. He argued that animals—including insects, rodents, and humans—use internal representations of number to guide behavior, such as tracking discrete events or estimating rates and durations. This capacity allows organisms to optimize behavior in dynamic environments, for example, by assessing the richness of a food patch or deciding when to abandon it (Gallistel, 1990). Gallistel's work also highlighted the computational efficiency of numerical encoding, proposing that it is an evolutionarily conserved mechanism for integrating and comparing magnitudes. While Gallistel did not specifically address plants, the same logic applies: Numerical representations can support accurate estimations of rate and probability—factors such as herbivore encounters, rainfall, and sunlight exposure—which are highly relevant to plant survival and reproductive success.

Building on this foundation, Dehaene (2011) introduced the concept of the ANS, an evolutionarily ancient and nonverbal mechanism for representing numerical quantity. His research has demonstrated that both human and nonhuman animals share the ability to estimate quantities approximately, with performance governed by Weber's law—discrimination between two quantities depends on their ratio rather than their absolute difference (Dehaene, 2011). He argued that the ANS supports a wide range of adaptive behaviors, including selecting the larger of two food patches, avoiding larger groups of predators, and managing social group sizes. Given the widespread presence of ANS‐like processing across the animal kingdom, it is plausible that plants also possess analogous mechanisms for encoding quantity, even in the absence of a nervous system.

Both Gallistel and Dehaene underscore that numerical cognition is not merely a symbolic capacity unique to humans but a fundamental and evolutionarily conserved method for representing discrete magnitudes. This system allows for generalization, comparison, and decision‐making across varied ecological contexts. From this perspective, the adaptive value of enumeration lies in its flexibility, abstraction, and efficiency—enabling organisms to respond appropriately to quantitative features of their environments, including novel or unpredictable situations.

Trewavas (2003) has further advanced the plausibility of enumeration in plants by outlining the biochemical basis for information processing in plant cells. He observed that many plant metabolic pathways support logic‐gate‐like behavior, including the implementation of functions such as AND, OR, and NOR. Proteins serve as key computational elements, and with roughly 1000 protein kinases present in both plants and animals, plants have access to rich regulatory networks. These include mechanisms for control, switching, and dynamic feedback—both positive and negative. Such metabolic systems can be likened to simple neural architectures or artificial neural networks, functioning as arrays of on/off switches with the capacity for complex information processing. These properties suggest that the biochemical machinery required for enumerative computation is present in plant cells, supporting the hypothesis that plants can encode and act on enumeration information in adaptive ways.

## 6 Plant and animal encoding of timing information

In the study we present here, plants were presented with a sequence of lighting events according to a particular schedule. Our hypothesis is that plants are capable of encoding the number of light‐on and light‐off events that have been presented, but that number was inescapably correlated with time. More time was always associated with more lighting events. As such, it is critical to consider the detailed literature on how plants encode temporal information. Ultimately, as with animal studies of enumeration, our goal was to see if plants are still capable of representing enumeration after effects of temporal correlations on behavior have been discounted.

### 6.1 Animal encoding of time

Research across many species has demonstrated that animals can encode and utilize time intervals to guide behavior, often with remarkable precision. This ability is central to many adaptive behaviors, such as foraging, mating, and predator avoidance. An influential model in this domain is the scalar expectancy theory (SET) proposed by Gibbon, Church, and Meck (1984), which posits that animals use a pacemaker‐accumulator mechanism to track elapsed time. In this model, pulses generated by an internal pacemaker are accumulated and stored in memory, where they are compared with temporal information from past experiences. A key prediction of SET is the “scalar property of variance”—that is, the standard deviation of an animal's timing responses scales proportionally with the interval being timed, a pattern supported by extensive empirical evidence (Church & Gibbon, 1982).

Buhusi and Meck (2005) provided an influential synthesis of behavioral and neurobiological findings on interval timing. They emphasized that interval timing involves distributed neural circuits—particularly cortico‐striatal loops modulated by dopamine—and that timing is not localized to a single “internal clock.” Their work highlighted the integration of timing with broader cognitive functions such as attention, working memory, and decision‐making. They proposed that timing reflects emergent properties of neural networks rather than relying on a central, dedicated mechanism. Church, Meck, and Gibbon (1994) showed that rats can time multiple intervals concurrently, suggesting parallel processing of temporal information.

Supporting evidence for animals’ timing capabilities has come from a wide array of species and paradigms. For example, Roberts (1981) demonstrated that pigeons and rats could accurately discriminate between short and long intervals using “fixed‐interval” and “peak‐interval” procedures. In the fixed‐interval procedure, an animal is trained to perform a specific action—such as pecking a key or pressing a lever—to receive a reward, but the reward becomes available only after a fixed amount of time has passed since the last reinforcement. Over time, animals learn to withhold responding early in the interval and increase response rate as the fixed time approaches. This generates a characteristic “scalloped” pattern of responding that reflects an emerging sense of the passage of time. The peak‐interval procedure is a refinement of the fixed‐interval method that allows researchers to more directly measure the temporal dynamics of internal time estimation. In this procedure, animals are first trained using a fixed‐interval schedule (e.g., press a lever after 30 s to receive food). Then, probe trials are introduced in which the reinforcement is omitted, and the stimulus remains present well beyond the trained interval (e.g., 90 s). During these non‐reinforced probe trials, animals continue to respond, but the rate of responding varies over time. The key observation is that the response rate typically forms a Gaussian‐like distribution centered around the trained interval (e.g., highest responding around 30 s) and then declines. This peak response—the point of highest response frequency—reflects the animal's internal estimate of the trained duration. The fact that responses cluster around a specific time point, even in the absence of reinforcement, strongly supports the idea that animals possess an internal clock or timing mechanism that tracks the passage of time with some precision.

Studies with primates also show advanced temporal capabilities. Merchant, Pérez, Zarco, and Gámez (2013) demonstrated that neurons in the rhesus macaque medial premotor cortex exhibit interval‐specific tuning across different timing tasks, suggesting a generalized neural mechanism for encoding time intervals in the primate brain. Moreover, research with insects such as bees has shown that they can associate rewards with time of day, indicating a form of interval and circadian timing (Boisvert & Sherry, 2006).

Gallistel and Gibbon (2000) argued for the ubiquity of quantitative representations (including time) in animal cognition, suggesting that temporal encoding is part of a broader symbolic processing system used for decision‐making and learning. Their model extends scalar timing by proposing that such quantitative representations underlie a wide range of cognitive tasks, from navigation to reward prediction.

In sum, converging evidence across taxa and methods supports the view that animals encode time intervals through mechanisms that are both behaviorally precise and neurally distributed. Theories such as SET and its successors have helped establish interval timing as a fundamental, evolutionarily conserved aspect of animal cognition.

### 6.2 Plant encoding of time

Research over the past several decades has produced growing evidence that plants possess mechanisms for tracking time and encoding interval duration, enabling them to coordinate physiological and behavioral responses to environmental cues. This capacity manifests in at least two broad forms: circadian rhythms and interval timing mechanisms that operate on shorter, non‐circadian scales.

The most thoroughly studied form of plant time tracking is the circadian rhythm, an endogenous clock that governs a wide array of biological processes according to an approximately 24‐h cycle. Many plants spread their leaves and orient them toward the sun when it rises in the morning. Classic work by Bünning (1956) demonstrated that plants produce these same rhythmic leaf movements even if they are placed in constant darkness, suggesting these movements are driven by an internal clock rather than being a stimulus‐driven response. Subsequent molecular biology studies have shown that plant circadian systems are entrained by light and temperature cues and involve a complex transcription–translation feedback loop, similar in architecture to those in animals (Harmer et al., 2000). These clocks coordinate photosynthesis, stomatal opening, growth, and flowering in alignment with daily environmental cycles.

Beyond circadian timekeeping, plants appear capable of encoding and responding to time intervals of shorter duration. Gagliano et al. (2014) trained *M. pudica* plants to associate a repeated drop stimulus with no harm. Over repeated trials, plants ceased closing their leaves in response—indicating habituation—and retained this learning for several days. Crucially, the retention was dependent on the regularity of the stimulus, suggesting an underlying timing mechanism sensitive to interval structure.

Another line of evidence comes from the work of Appel and Cocroft (2014), who found that *Arabidopsis* plants can detect and respond to the specific temporal pattern of herbivore feeding vibrations. Plants primed with feeding vibrations produced higher levels of defensive chemicals compared to controls, indicating temporal sensitivity to vibration stimuli.

Thellier and Lüttge (2013), based on a review of many studies, proposed that plants possess “memory windows” for physiological responses that reflect sensitivity to temporal patterns in environmental inputs. The memory window is a temporally limited period during which a plant exhibits heightened sensitivity to the recurrence of particular environmental stimuli. They propose that memory in plants involves two phases. During the “induction phase,” some stimulus that alters the physiological state of the plant occurs. During the subsequent memory window phase, the plant remains sensitive to a second stimulus. If a second stimulus occurs within this window, the plant can integrate the temporal relationship between the stimuli, leading to an altered or enhanced physiological response. If the second stimulus falls outside this window, no integration or memory trace occurs. Some memory traces may fade if not reinforced, while others become consolidated and persist.

The memory window concept is largely theoretical, but it comports with several lines of research. For instance, Monshausen, Bibikova, Weisenseel, and Gilroy (2009) found that the responses of *Arabidopsis* roots to mechanical touch stimulation are modulated by prior exposure to light, but only when that light is delivered within a particular time window. Touching the *Arabidopsis* roots activates calcium signaling and reactive oxygen species (ROS) production. In their experiment, Monshausen et al. exposed some plants to blue light prior to the touch stimulus, while control plants were kept in darkness. Calcium imaging and fluorescent ROS indicators were used to monitor physiological responses in real time. Mechanical stimulation induced rapid calcium influx and ROS production in root cells—well‐established markers of mechanosensory response. Pre‐exposure to blue light significantly enhanced both the calcium and ROS responses to mechanical stimulation. The enhancement was time‐sensitive—only occurring when the light preceded the mechanical stimulus within a particular temporal window. The plant response to one stimulus (touch) was modulated by the prior occurrence of another stimulus (light)—and the effect depended on the temporal proximity of the two stimuli. In the context of the memory window concept, the plant altered its mechanosensory physiology based on its recent sensory history, demonstrating a primitive form of temporal integration or associative modulation. While this is not associative learning in the strict behavioral sense (no conditioning trial structure or behavioral outcome was assessed), it provides evidence for time‐sensitive physiological “priming” in plants—closely aligning with Thellier and Lüttge's concept of a memory window.

In summary, a growing body of research suggests that plants can track both circadian and non‐circadian time intervals, allowing them to optimize behavior such as learning, foraging, and defense in dynamic environments. This capacity underscores the broader view that temporal cognition is not limited to organisms with nervous systems.

## 7 Plant movement patterns and terminology

A wide range of evidence suggests that both plants and animals are capable of encoding quantity information. Research also suggests that animals are further capable of using enumeration information, abstracting number information from sensory characteristics of stimuli. There is, however, little to no evidence for enumeration‐based information processing in plants. A range of evidence suggests that this is computationally and evolutionarily plausible. There is no clear reason to expect that adaptively valuable enumeration processing would have arisen only in the animal kingdom.

The hypothesis of the study we present here is that plants are capable of enumeration‐based information processing. We set out to test this with the *M. pudica* plant by assessing its movement responses when exposed to patterns of light and dark. Before we proceed with the details of the study, we consider the different types of movements that plants exhibit, along with the terminology that is commonly used to describe those movements. Plants, despite being rooted in place, exhibit various types of movement in response to stimuli. These movements can be broadly categorized into “tropic” and “nastic” movements (Satter & Galston, 1981).

Tropic movements are directional movements where the plant grows either towards or away from a stimulus. Most plants exhibit phototropism, in which shoots and leaves generally grow toward light (positive phototropism), while roots typically grow away from light (negative phototropism). Geotropism, movements in response to gravity, describes roots growing downward (positive geotropism), while shoots grow upward (negative geotropism). Roots grow toward water sources, exhibiting positive hydrotropism. Some plants exhibit chemotropism, growth toward or away from a chemical concentration gradient. Some plants exhibit thigmotropism, movement in response to touch or contact with a solid object. Tendrils of climbing plants wrapping around a support are a classic example. Thermotropism (movement in response to temperature changes) and aerotropism (movements in response to air) have also been documented (Satter & Galston, 1981).

Nastic movements are typically defined as non‐directional movements in response to a stimulus. The direction of these movements is independent of the stimulus direction. Photonasty is movement in response to changes in light intensity. Flowers opening during the day and closing at night are an example. Thermonasty is a movement in response to temperature variations, also often seen in the opening and closing of flowers. Non‐directional responses to gravity, chemical stimuli, and water are defined, respectively, as geonasty, chemonasty, and hydronasty (Satter & Galston, 1981).

*Mimosa pudica* plants, on which the current study focuses, exhibit both “nyctinastic” and “thigmonastic” movements. Nyctinastic movement in plants refers to rhythmic, non‐directional movements that occur in response to the daily light–dark cycle, typically driven by the plant's internal circadian clock. These movements are most commonly seen in the opening and closing of leaves, petals, or other organs in response to changes in light intensity and temperature associated with day and night. For example, in *M. pudica*, the leaves fold inward at night and reopen during the day—a classic nyctinastic response. Importantly, the direction of the movement is independent of the direction of the stimulus, which distinguishes it from tropic movements. Nyctinasty is thought to serve adaptive functions such as reducing water loss, protecting tissues from cold night temperatures, or minimizing herbivore exposure during vulnerable periods.

Nyctinasty is strongly associated with movements around sunrise and sunset, but the plants do not remain stationary in between these events. As we will describe in our current study results, *M. pudica* sways laterally throughout the day and night hours. The amount of this sway varies according to a daily cycle that is influenced by daily lighting cycles. Our data suggest that it is also influenced by the number of lighting events that have occurred in the recent past.

Thigmonastic movement (also called seismonastic movement) is a rapid, non‐directional response to mechanical stimuli such as touch, vibration, wind, or sudden shock. When touched, *M. pudica* leaflets quickly fold together and their supporting stems droop—often within 1–2 s. Thigmonastic movements are thought to provide protective benefits, such as deterring herbivores or minimizing physical damage from environmental factors (Satter & Galston, 1981). *Mimosa pudica* is well known for these rapid responses to touch stimuli, which have led it to be known as the “touch me not” or “shy” plant.

The study presented here focused on how a sequence of light and dark events influenced the amount of nyctinastic movement exhibited by the plants. Future studies could similarly assess these hypotheses with the more well‐known thigmonastic movements.

## 8 Overview of the study

**Fig. 1:**
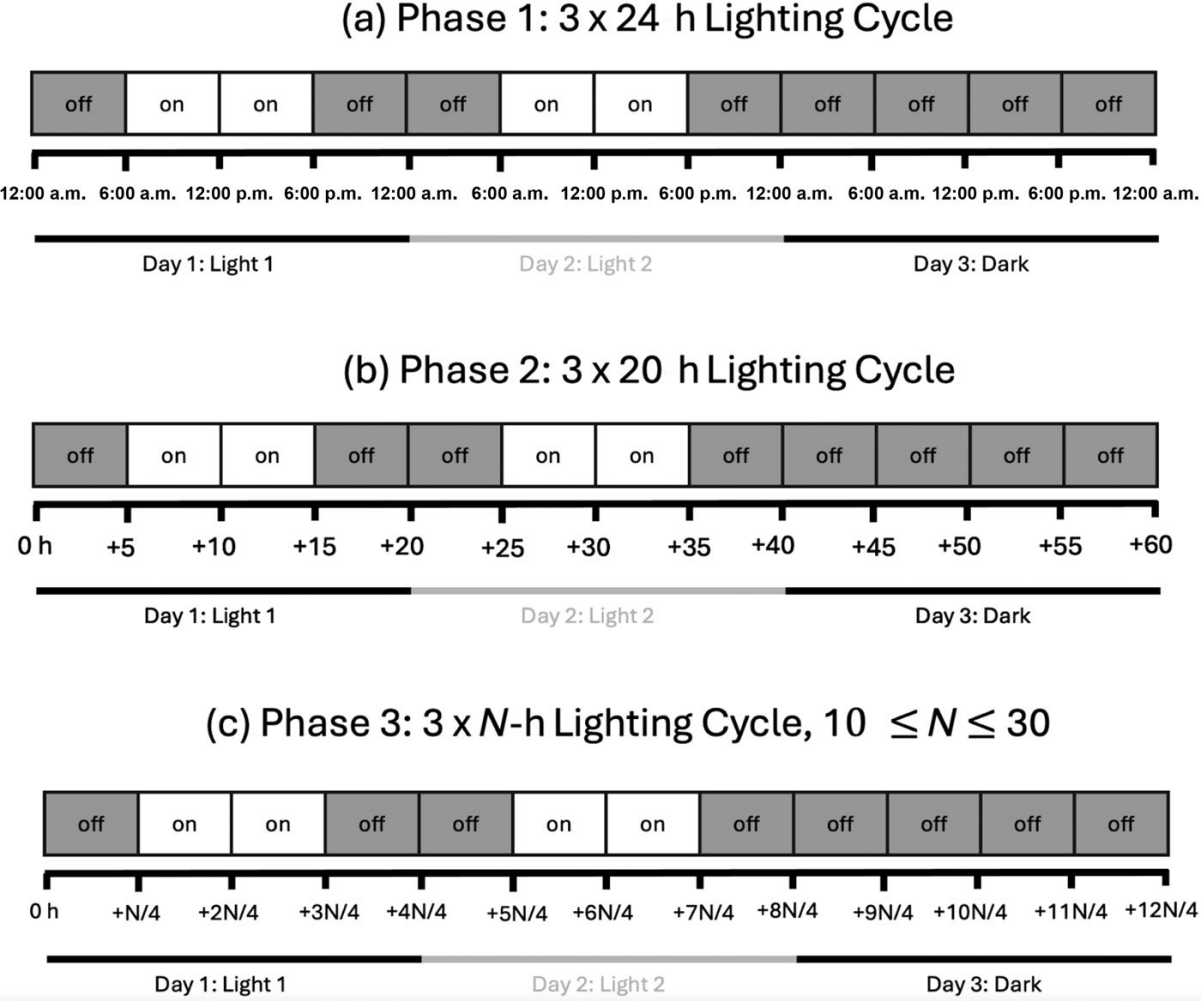
Timing of plant lighting across the three phases of the study. In Phase 1, we repeatedly presented plants with a 72‐h, 3‐day cycle. Lights turned on for 12 h per day for 2 days, followed by a third day in which the lights remained off. In Phase 2, we shortened the cycle from 72 to 60 h (24‐h days to 20‐h days). In Phase 3, a day length was randomly selected at the start of each light–light–dark cycle, between 10 and 32 h. The light–light–dark pattern was presented based on this day's length.

We raised a group of *M. pudica* plants in a light‐attenuating tent, such that the only substantial source of illumination came from two arrays of overhead grow lights. These grow lights were turned on and off according to a pre‐planned schedule. In the *24‐h* phase of the study, the lights would turn on at 6:00 a.m. and remain on until 6:00 p.m., when they would turn off. The lights would follow this schedule for 2 days, after which the lights remained off during the third day (Fig. 1). This cycle was repeated for 74 days. After 15 days, the plants exhibited nyctinastic movement patterns that followed this *Light1‐Light2‐Dark* cycle. Specifically, between midnight and 6:00 a.m., during *pre‐light* hours, the plants moved more on days when the lights would turn on than on days when darkness continued through the day. The nyctinastic movement levels seemed to anticipate the presence or absence of future lighting conditions.

Were the plants enumerating the light events or tracking the passage of time? Since the day length was always 24 h, the passage of time was confounded with the number of lighting events. To differentiate between time and number, we changed the day length from 24 to 20 h. In the *20‐h* phase of the study, the lights would turn on for 10 h of every 20‐h period. After two 10‐h light events, the lights would remain off for a full 20 h. Almost immediately, the plants seemed to exhibit a pattern that was linked to the number of light events, not the number of hours elapsed.

In the third, *random‐hour* phase of the study, the length of light exposure varied with every three‐event cycle. At the onset of this phase, a “day duration” between 10 and 32 h was randomly selected. The lights turned on for half of this day duration and then off for the second half. This was repeated a second time, after which the lights remained off for a full day duration. In this condition, time was no longer an effective predictor of when the lights would stay off for an extended period. The enumeration information, however, remained an effective predictor. After two light exposure events, separated by darkness events, it could be inferred that an extended period of darkness would follow. Plant nyctinastic movement patterns operated in accord with this inferential process.

For all three study phases, we analyzed the data using repeated‐measures anovas conducted on the time series of 3‐day cycles for the plant group (Phase 1: 23 cycles; Phase 2: 6 cycles; Phase 3: 30 cycles).

## 9 Phase 1: 24‐h days–2 days “on,” 1 day “off”

We presented plants with a predictable cycle of light exposures for 74 days to see if the plants would respond in ways that implicitly track that cycle. For the first 2 of every 3 days, grow lights would turn on for 12 h and turn off for 12 h. On the third day, the lights would remain off, resulting in 36 continuous hours of darkness. By analyzing patterns of nyctinastic movement produced by the plants, we assessed the extent to which the plants are capable of learning this pattern.

### 9.1 Method

We obtained *M. pudica* seeds from RDR Seeds (Garden City), soaked them in warm water (approximately 60°C) for 20 min and then planted them in 18 plastic cups (9.2 cm diameter, 11.7 cm height) filled with equal amounts of potting soil (Miracle Gro) and vermiculite (Good Earth Organics). We planted five seeds in each cup and watered them every 2–5 days. After 30 days in an indoor space with partial direct sunlight, temperatures 19–24°C and 60%–70% ambient humidity (measured with ThermoPro TP359, ThermoPro), the plants were approximately 10 cm in height.

We moved the plants to a black, light‐attenuating storage tent (2.4 × 1.8 m length and width; 1.8 m height, Advance Outdoor Solutions). Two LED grow‐light panels (Mars‐TS‐600W‐FBA, Mars Hydro) were hung above the plants, 1 m above the table surface. Plants were placed close together, on plastic trays. Temperature and humidity were 19–24°C and 60%–70% throughout the indoor study process.

A Raspberry Pi (model 3B+, Raspberry Pi Foundation) controlled lighting and recorded time‐lapse video of the plants using an infrared‐equipped camera (Jun Sun Electronic Technology). The camera module captured a 1920 × 1080 pixel image of the plants every 20 s. We encoded these into mpeg video recordings using open‐source software (FFmpeg, ffmpeg.org). For each 24‐h cycle, we produced a 4320‐frame video.

### 9.2 Light cycle pattern

Lighting followed a 72‐h cycle. For the first 48 h of the cycle, between 12:00 and 6:00 a.m., the lights were off. From 6:00 a.m. to 6:00 p.m., the lights were on. From 6:00 p.m. until 12:00 a.m., the lights were off. For the last 24 h of the 72‐h cycle, the lights would remain off. Put more simply, we provided the plants with 2 days of 12 h on/12 h off light. On the third day, the plants remained in darkness. Hereafter, we refer to these three 24‐h *lighting* conditions intervals as light Day 1 (*Light1*), light Day 2 (*Light2*), and dark day (*Dark*).

### 9.3 Video processing and analysis

Video processing was performed using Matlab software (Mathworks). JPEG images are defined in terms of red, green, and blue values for each pixel, with each ranging from 0 to 255. We restricted our analysis to the green channel. For each frame of each video, we calculated the average amount of change in these pixel values, as compared with the prior video image using the Matlab “diff” function. In general, larger amounts of plant movement produce higher levels of average pixel change.

When the grow lights turned off, the camera automatically switched to infrared recording mode, activating infrared light sources. These lighting changes resulted in one frame in which a very large pixel change occurred. These frames, along with the frame before and after this time, were excluded from the analysis. The mpeg video compression process makes use of periodic “key frames,” which resulted in large, illusory change values, which were also excluded from the analysis. Large change values were also produced when the plants were watered. When we sprayed the plants, the leaves would close and then reopen for reasons unrelated to the light cycle. We thus removed frame values collected during watering and during the 70 min after watering was completed. Three of the 74 days were excluded due to computer image collection failures.

All videos and Matlab analysis scripts are available online. See the Data Availability Statement for access information.

### 9.4 Use of pixel change as a measure of plant response

In this study, plant responses were quantified by measuring the average pixel change between successive video frames. Nyctinastic movements clearly contributed to these changes, as the motion of stems and leaves caused alterations in their projected positions on the video image. As the plant organs shifted, previously occupied pixels became unoccupied and vice versa. Although this measure reliably captured movement, it is important to acknowledge that other factors may have influenced the observed pixel change. For instance, while we did not detect substantial variation in leaf coloration, such changes could also affect pixel values and thus the movement metric.

Multiple components of the plant contribute to its observable movements. However, our pixel‐based measure does not differentiate which specific structures were responsible for the changes observed. Movements in *M. pudica* are mediated by highly specialized anatomical and physiological mechanisms localized in the pulvinus—a joint‐like swelling found at the base of both petioles and leaflets. Each pulvinus contains two layers of motor cells, adaxial (upper) and abaxial (lower), which regulate movement through differential changes in turgor pressure. Upon stimulation, these cells undergo rapid ionic fluxes—particularly involving potassium (K⁺), chloride (Cl⁽), and calcium (Ca^2^⁺)—that drive osmotic shifts in water content and result in reversible volume changes (Satter & Galston, 1981b). Some of these movements are triggered by action potentials and associated electrical signaling, while others rely on slower, ion‐mediated transport processes. Our pixel‐based measures do not enable us to differentiate among these subsystems. Any or all of these movement‐generating systems could have produced the patterns we report here.

Although the plants were not manually stimulated during the recorded segments analyzed in this study, incidental contact between plants periodically caused thigmonastic responses occurring alongside the nyctinastic movements. The average pixel change metric does not distinguish between nyctinastic and thigmonastic motor activity, nor does it isolate the contributions of distinct physiological mechanisms.

Nevertheless, our results support the inference that at least one of these movement‐generating systems may encode enumeration‐relevant information. Future research may benefit from identifying the particular motor mechanisms responsible for this capacity. The current study contributes to the field by presenting evidence consistent with enumeration‐based information processing at the system level, as inferred from a non‐invasive, image‐based analysis of movement.

### 9.5 Time series assessment of the plant group: Details and limitations

Although we report the number of cups and seeds used in the study, the plants were not analyzed individually or by cup. Instead, the plants were grown in close proximity and often made physical contact with neighboring plants, both within the same cup and across adjacent cups. Our analysis focused on the collective behavior of the entire plant group, examined over time and across varying lighting conditions. This group‐based approach aligns with established practices in behavioral science, where detailed insights are often derived from studies with limited or non‐independent subjects. For example, Brannon and Terrace (1998), whose work significantly informs thinking in this domain, based their findings on experiments with only two monkeys.

Future studies involving a large number of plants grown in isolation may yield important insights into individual‐level processing, but examining plants as a collective system is also crucial. In the wild, *M. pudica* plants often grow in groups. Plants are not passive or solitary organisms; they engage in meaningful social interactions and even cooperative behaviors (Karban, 2008). It is plausible that the effects we observed in this study would not have emerged in the absence of such plant‐plant interactions. Further research will be necessary to determine whether enumeration‐based processing is uniformly distributed across individuals or confined to a subset of plants. For instance, it is possible that only some plants contributed to the observed group‐level effects, while others did not engage in such processing at all.

Nonetheless, our current findings suggest that at least some members of the group exhibited evidence of enumeration‐based information processing. As Ramachandran and Blakeslee (1998) argue, single‐case observations can have powerful implications for scientific understanding: to prove that pigs can talk, you only need one pig. Likewise, our study provides an initial demonstration that such numerical processing may be possible in plants, even if only a subset of individuals is responsible for the observed effects.

The study included a fully within‐subjects design, which we analyzed using repeated‐measures ANOVA: *Time‐of‐day* (three levels: *pre‐light*, *light‐on*, *post‐light*), *Day* condition (three levels: *Light1*, *Light2*, *Dark*), *Cycle* number (23 levels: cycles 1–23). For *Day* and *Time‐of‐day* repeated‐measures factors, the numerator degrees of freedom correspond to the number of levels minus one (*Day*, 2 df; *Time‐of‐day* 2 df). *Cycle* number was treated as a covariate, resulting in 23 − 2 = 21 df. When these factors are combined in interactions, their numerator df multiply.

Day×Cycle→2×21=42df

Time−of−day×Cycle→2×21=42df

Day×Time−of−day×Cycle→2×2×21=84df

*F*(2, 42) corresponds to a factor or interaction with 2 numerator df and 42 denominator df. *F*(4, 84) corresponds to a factor or interaction with 4 numerator df and 84 denominator df. These denominator dfs reflect the error terms associated with those within‐subject effects: df_error_ = (*n*−1) × df_factor_. For *F*(2, 42): (*n*−1) × 2 = 42 → n = 22. For *F*(4, 84): (*n*−1) × 4 = 84 → n = 22.

### 9.6 Results and discussion

**Fig. 2:**
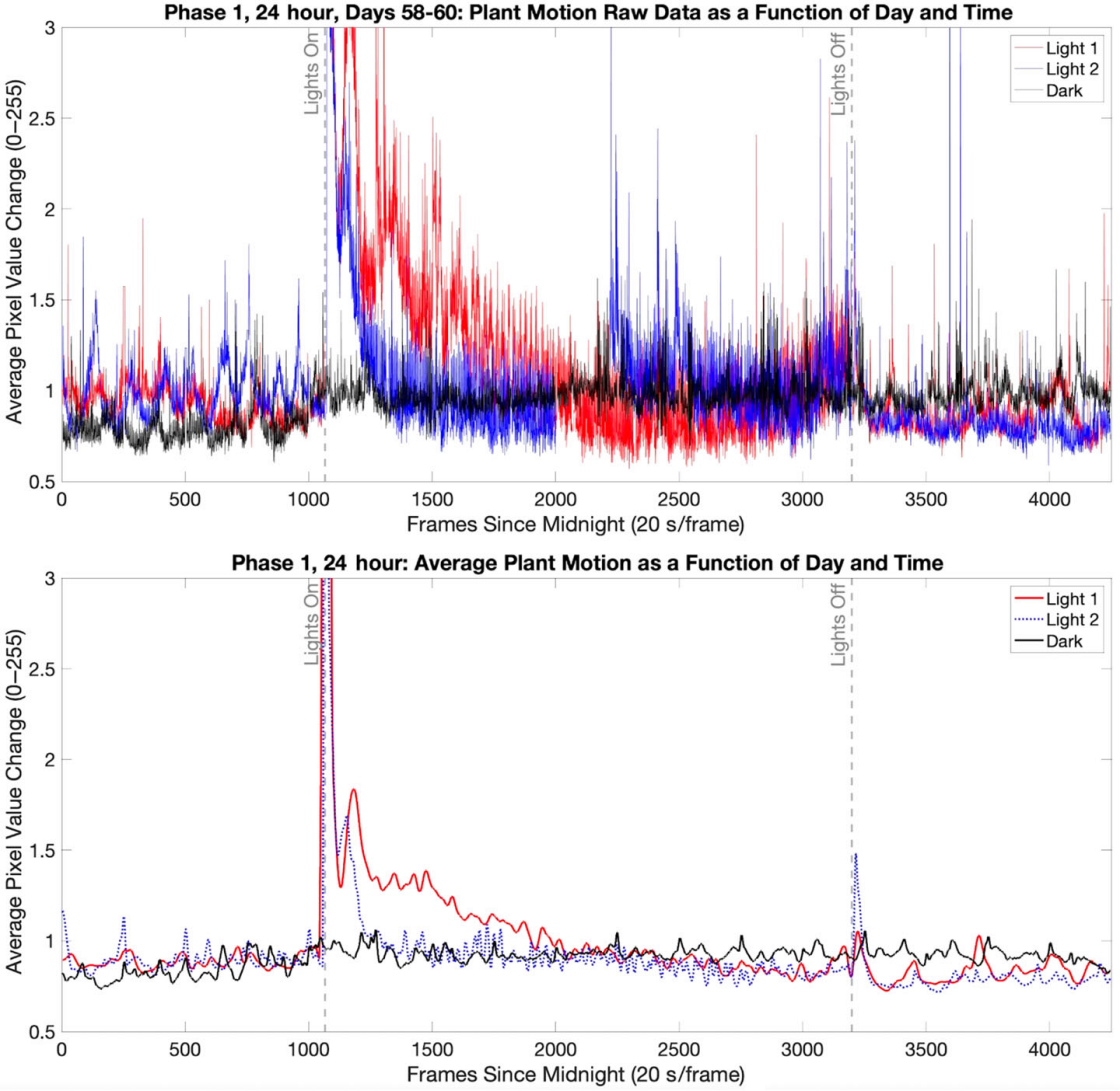
Plant movement levels exhibited a clear circadian pattern that was moderated by the *Light1‐Light2‐Dark* lighting cycle. Raw data from Days 58 to 60 (Cycle 20) are presented in the top panel. Data in the bottom panel are average values from study Phase 1, smoothed with a Gaussian filter with a window of 50 frames.

Plant motion exhibited a clear 24‐h periodicity (Fig. 2). During the *pre‐light* interval of the day, the plants exhibited relatively low levels of motion. During the *light‐on* interval in the middle of the day, the plants exhibited a spike in movement at the time of light onset, followed by motion levels which were higher than those observed during dark hours. When the lights turned off, the plants exhibited another spike in motion as the leaves tended to close and angle downward. During the *post‐light* interval of the day, the plants returned to relatively low levels of motion.

We used repeated measures ANOVA to explore motion levels at each of the three *times‐of‐day* (*pre‐light, light‐on, post‐light*), with each of the three *day* conditions (*Light1, Light2, Dark*), across the 23 3‐day *cycles* for which we collected usable data. Group descriptive statistics and the full ANOVA output table are presented in Tables 1 and 2. Significant two‐way interactions were apparent between *day* condition and *cycle* number (*F*(2, 42) = 5.5, *p* = .007, *Ƞ* ^2^ *_p_* = 0.21) and between *time‐of‐day* and *cycle* number (*F*(2, 42) = 4.4, *p* = .02, *Ƞ* ^2^ *_p_* = 0.17). A significant three‐way interaction was present between *day, time‐of‐day*, and *cycle* number, *F*(4, 84) = 3.0, *p* = .024, *Ƞ* ^2^ *_p_* = 0.12. These interactions strongly suggest that the plants’ response to lighting events changed substantially over the course of the study.

**Table 1:** Study Phase 1, 24‐h day duration: Descriptive statistics for motion levels

Day Condition/Time‐of‐Day	*N*	*Mean*	*SEM*
L1 Pre‐light	23	0.890	0.022
L2 Pre‐light	23	0.898	0.017
D1 Pre‐light	23	0.840	0.017
L1 Light‐on	23	1.035	0.044
L2 Light‐on	23	0.914	0.056
D1 Light‐on	23	0.929	0.014
L1 Post‐light	23	0.830	0.014
L2 Post‐light	23	0.784	0.012
D1 Post‐light	23	0.918	0.024

**Table 2:** Study Phase 1: 24‐h day duration, repeated measures anova

Tests of Within‐Subjects Effects						
Source	Sum of Squares	df	*Mean* Square	*F*	*p*	*η* ^2^_*p*_
Time‐of‐day	0.0439	2	0.02193	1.05	.359	0.048
Time‐of‐day × cycle	0.1838	2	0.09188	4.39	.019	0.173
Residual	0.8784	42	0.02091			
Day condition	0.0801	2	0.04006	3.46	.041	0.141
Day condition × cycle	0.1284	2	0.06419	5.54	.007	0.209
Residual	0.4867	42	0.01159			
Time‐of‐day × day condition	0.0230	4	0.00576	0.44	.779	0.021
Time‐of‐day × day condition × cycle	0.1556	4	0.03889	2.98	.024	0.124
Residual	1.0972	84	0.01306			

Tests of Between‐Subjects Effects						
Source	Sum of Squares	df	*Mean* Square	*F*	*p*	*η* ^2^_*p*_
Cycle	0.0379	1	0.0379	1.17	.291	0.053
Residual	0.6778	21	0.0323			

*Note*. Type III sums of squares were used for all effects.

**Fig. 3:**
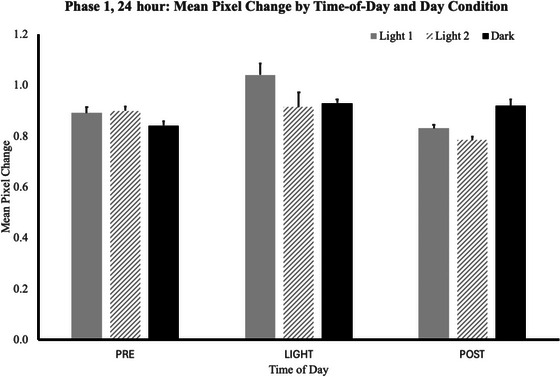
For study Phase 1 (24‐h days), mean pixel change values are shown for each of the three *time‐of‐day* intervals (*pre‐light*, *light*, and *post‐light*), for each of the *day* conditions (*Light1*, *Light2*, and *Dark*). During the *pre‐light* interval, regardless of *day* condition, plants were always in the dark and had been so for at least 6 h. The plants moved more during the *pre‐light* time on days when light could be expected than on days when darkness followed.

To interpret these interactions, we employed a simple effects analysis, examining how each *day* condition and *cycle* number influenced the movement levels at each of the three *time‐of‐day* levels (Fig. 3). The full ANOVA output is presented in Table 3.

**Table 3:** Study Phase 1: 24‐h day duration, pre‐light time‐of‐day simple effects repeated measures ANOVA

Tests of Within‐Subjects Effects						
Source	Sum of Squares	df	*Mean* Square	*F*	*p*	*η* ^2^_*p*_
Day condition	0.0263	2	0.01316	2.17	.127	0.094
Day condition × cycle	0.0808	2	0.04040	6.67	.003	0.241
Residual	0.2545	42	0.00606			

Tests of Between‐Subjects Effects						
Source	Sum of Squares	df	*Mean* Square	*F*	*p*	*η* ^2^_*p*_
Cycle	0.0217	1	0.02174	2.44	.133	0.104
Residual	0.1872	21	0.00891			

*Note*. Type III sums of squares were used for all effects.

The most meaningful simple effects level is the *pre‐light* interval, between 12:00 and 6:00 a.m. During this interval, regardless of *day* condition, the plants were always in the dark and had been so for at least 6 h. During the *pre‐light* time, the plants exhibited lower levels of movement in the *Dark day* condition than in the *Light1* or *Light2* conditions. A highly significant interaction between *day* condition and cycle number emerged, *F*(2,42) = 6.7, *p* = .003, *Ƞ* ^2^ *_p_* = 0.24 (*Light1 mean* = 0.89, *SEM* = 0.02; *Light2 mean* = 0.90, *SEM* = 0.02; *Dark mean* = 0.84, *SEM* = 0.01).

During the *light* time of the day, a significant interaction between *day* condition and *cycle* number emerged, *F*(2,42) = 3.9, *p* = .028, *Ƞ* ^2^ *_p_* = 0.14 (*Light1 mean* = 1.04, *SEM* = 0.04; *Light2 mean* = 0.91, *SEM* = 0.06; *Dark mean* = 0.93, *SEM* = 0.01). More movement was apparent in the *Light1* condition than for *Light2* or *Dark* conditions during this interval.

During the *post‐light* time of the day, no significant differences or interactions emerged (*Light1 mean* = 0.83, *SEM* = 0.14; *Light2 mean* = 0.78, *SEM* = 0.12; *Dark mean* = 0.92, *SEM* = 0.02). More movement was observed in the *Dark* condition than for *Light1* or *Light2* conditions.

**Fig. 4:**
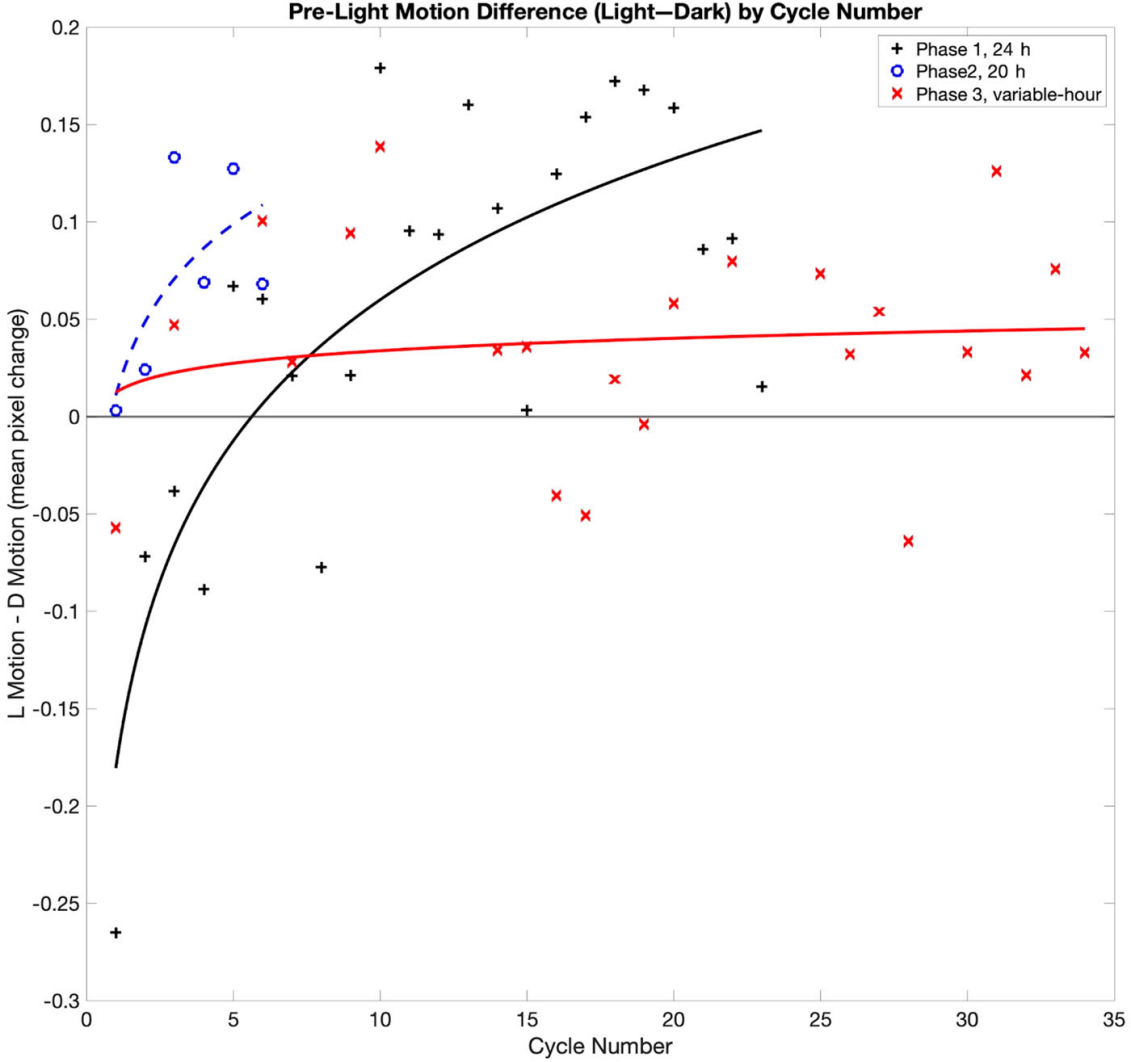
Differences between plant movements during the pre‐light time‐of‐day of the Light and *Dark* conditions. For each 3‐day *cycle* of the study, the average *Dark* motion level was subtracted from the average of the two *Light* motion levels: [(*Light1* + *Light2*)/2] − *Dark*. Positive values indicate that during the *pre‐light* intervals, the plants moved more in the hours leading up to the onset of the light and less on days in which there was no light. Conversely, negative values indicate that during the *pre‐light* intervals, the plants moved less in the hours leading up to the onset of the light and more on days in which there was no light. Phase 1 of the study used a 24‐h day length. Plants altered their movement pattern during the course of the study, producing relatively less movement on days in which lights would not turn on. In study Phase 2, the plants continued this pattern even when the day length was shifted to 20 h. The pattern also generalized to Phase 3, in which the day length was randomly varied after each *Light1‐Light2‐Dark* cycle, when the day lengths were between 12 and 24 h.

When a low level of plant motion was present during the *pre‐light* time of day, we can infer that the lights would not turn on at the end of that interval. It is especially notable that this trend was not present at the beginning of the study. During the first six 3‐day cycles, activity during the *Dark* days was actually higher than that observed on *Light1* or *Light2* days. Fig. 4 depicts the difference in observed motion during the *pre‐light* time prior to lighted days and dark days:

$$\left(Light1+Light2\right)/2-Dark$$

as a function of *cycle* number. This difference is well‐fit by a logarithmic function, with an *r* ^2^ value of 0.63.

$$\mathrm{MotionDifference}=0.10^{*}log\left(\mathrm{cyclenumber}\right)-0.18$$

Over the course of 74 days, the plants shifted their nyctinastic movements in accord with the *Light1‐Light2‐Dark* sequence. The shift was apparent by day 15 of the study, after five repetitions of the *Light1‐Light2‐Dark* cycle.

On days when light was likely to turn on, the plants moved more during the hours prior to light onset. Conversely, on days when the light was unlikely, the plants moved less during those same hours. This trend was not present for the first 12 days of the study but emerged as a function of experience. If we observed Fig. 4 with an animal participant, we would likely describe it as characterizing a learning process. Logarithmic functions of this type have frequently been observed in classic and operant conditioning data, memory and forgetting functions, and a wide range of other paradigms (Sargisson & White, 2003; Stepanov & Abramson, 2008; White, 2001).

These results suggest that the plants were able to represent information about the number of light events that had been presented during the previous 48 h, but there is an alternative possibility. Time is highly correlated with the number of lighting events presented to the plants. Perhaps it was the passage of time, and not the number of lighting events, that influenced the movement behaviors of the plants. Many plants, including *M. pudica*, exhibit movements that follow a 24‐h, circadian rhythm (e.g., Calvo, Gagliano, Souza, & Trewavas, 2020). Clearly some element of the plants’ physiology tracks that passage of time. It could be that this mechanism encodes the passage of a 72‐h pattern. We know of no evidence that plants are capable of tracking 72‐h cycles, but it is possible that they do. If so, tracking the number‐correlated passage of time could have produced the patterns observed in the first phase of this study.

In order to assess the extent to which the plant movement cycles were driven by temporal factors, we changed the light cycle pattern from 24‐h days to 20‐h days and observed the changes in plant movement behaviors.

## 10 Phase 2: 20‐h days–2 short days “on,” 1 short day “off**”**

All of the plants used in Phase 1 of this study (24‐h days) continued immediately into Phase 2 (20‐h days). If the plants were tracking a 72‐h cycle and not enumerating the lighting events, they should have quickly shifted out of phase with the *Light1‐Light2‐Dark* cycle. Low levels or *pre‐light* motion would be expected to arrive 4 h later each day relative to lighting onset. This next phase of the study assessed whether the plants would need to re‐acquire the information gathered during the first 15 cycles of study Phase 1.

### 10.1 Method

The same plants used in Phase 1 continued into Phase 2. We used the same tent, lighting, image collection, and watering protocols. The only thing we changed was the timing of the lighting cycle. The “day” length was shortened to 20 h.

Lighting followed a 60‐h cycle (Fig. 1). For the first 5 h, the lights were off. They then turned on for 10 h, after which they turned off for the last 5 h of the 20‐h “day.” This 20‐h cycle repeated, after which the light remained off for 20 continuous hours. Put more simply, we provided the plants with two 20‐h days of 10 h on/10 h off light. On the third 20‐h day, the plants remained in darkness. We repeated this sequence for eight 3‐day *cycles*. Due to computer failures, the data from the final two 3‐day cycles were incomplete. We thus analyzed data from the initial six cycles.

We did not include a control group of plants that continued on with the 24‐h cycle. Our primary question was whether the plants would need to re‐learn the 20‐h day length or whether learning from Phase 1 would generalize into Phase 2. We are confident that had the plants continued on the 24‐h cycle, the pattern of performance would have continued to progress toward the asymptote suggested by the logarithmic function in Fig. 4. That said, it is possible that the plants were about to change their movement patterns. Our current design did not address this issue.

### 10.2 Design and analysis

The study included a fully within‐subjects design, which we analyzed using repeated‐measures ANOVA: *Time‐of‐day* (three levels: *pre‐light*, *light‐on*, *post‐light*), *Day* condition (three levels: *Light1*, *Light2*, *Dark*), *Cycle* number (six levels: cycles 1–6). For *Day* and *Time‐of‐day* repeated‐measures factors, the numerator degrees of freedom correspond to the number of levels minus one (*Day*, 2 df; *Time‐of‐day* 2 df). *Cycle* number was treated as a covariate, resulting in 6 − 2 = 4 df. When these factors are combined in interactions, their numerator df multiply.

Day×Cycle→2×4=8df

Time−of−day×Cycle→2×4=8df

Day×Time−of−day×Cycle→2×2×4=16df

*F*(2, 16) corresponds to a factor or interaction with 2 numerator df and 16 denominator df. *F*(4, 16) corresponds to a factor or interaction with 4 numerator df and 16 denominator df.

Note that we terminated this phase of the study after 18 days, that is, six repetitions of the *Light1‐Light2‐Dark* cycle. We chose to do this for two reasons. First, the goal of this phase was to test whether the plant motions would continue to follow the number of light events, even when the timing of those events changed. By Day 18, this result was clear. Second, we wished to test whether the plants would similarly generalize their movement patterns if we randomly changed the timing of the lighting events repeatedly. See study Phase 3 for details on this procedure.

### 10.3 Results and discussion

We again used repeated measures ANOVA to explore motion levels at each of the three *times‐of‐day* (*pre‐light, light‐on, post‐light*), with each of the three *day* conditions (*Light1, Light2, Dark*), across the six 3‐day *cycles* for which we collected usable data. Group descriptive statistics and the full ANOVA output table are presented in Tables 4 and 5.

**Table 4:** Study Phase 2, 20‐h day duration: Descriptive statistics for motion levels

‐Day Condition/Time‐of‐Day	*N*	*Mean*	*SEM*
L1 Pre‐light	6	0.834	0.031
L2 Pre‐light	6	0.863	0.016
D1 Pre‐light	6	0.778	0.015
L1 Light‐on	6	1.404	0.038
L2 Light‐on	6	0.997	0.029
D1 Light‐on	6	0.818	0.018
L1 Post‐light	6	0.815	0.017
L2 Post‐light	6	0.894	0.020
D1 Post‐light	6	0.849	0.013

**Table 5:** Study Phase 2: 20‐h day duration, repeated measures ANOVA

Tests of Within‐Subjects Effects						
Source	Sum of Squares	df	*Mean* Square	*F*	*p*	*η* ^2^_*p*_
Time‐of‐day	0.15564	2	0.07782	29.22	< .001	0.880
Time‐of‐day × cycle	0.00558	2	0.00279	1.05	.394	0.208
Residual	0.02131	8	0.00266			
Day condition	0.07801	2	0.03900	83.80	< .001	0.954
Day condition × cycle	3.09e−4	2	1.55e−4	0.33	.727	0.077
Residual	0.00372	8	4.65e−4			
Time‐of‐day × day condition	0.15273	4	0.03818	16.75	< .001	0.807
Time‐of‐day × day condition × cycle	0.01102	4	0.00276	1.21	.345	0.232
Residual	0.03647	16	0.00228			

Tests of Between‐Subjects Effects						
Source	Sum of Squares	df	*Mean* Square	*F*	*p*	*η* ^2^_*p*_
Cycle	8.69e−4	1	8.69e−4	0.05	.831	0.013
Residual	0.0668	4	0.0167			

*Note*. Type III sums of squares were used for all effects.

**Fig. 5:**
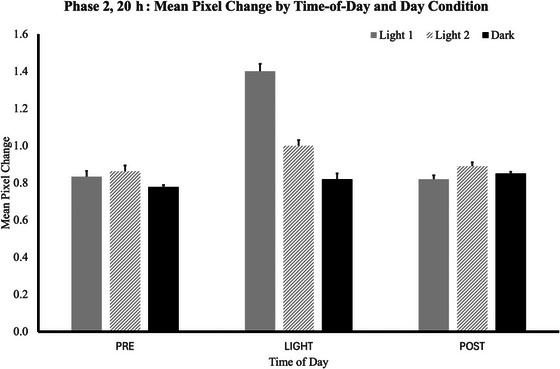
For study Phase 2 (20‐h days), mean pixel change values are shown for each of the three *time‐of‐day* intervals (*pre‐light*, *light*, and *post‐light*), for each of the *day* conditions (*Light1*, *Light2*, and *Dark*). During the *pre‐light* interval, regardless of *day* condition, plants were always in the dark and had been so for at least 5 h. The plants moved more during the *pre‐light* time on days when light could be expected than on days when darkness followed.

Many of the same patterns observed in Phase 1 of the study were again present (Fig. 5). The plants moved significantly more when the lights were on than during the dark periods of the day, producing a significant main effect of *time‐of‐day*, *F*(2, 8) = 29.2, *p* < .001, *Ƞ* ^2^ *_p_* = 0.88. Significantly more overall motion was observed in the *Light1* and *Light2* conditions than in the *Dark* condition. (*Light1 mean* = 1.0, *SEM* = 0.02; *Light2 mean* = 0.92, *SEM* = 0.02; *Dark mean* = 0.82, *SEM* = 0.02, *F*(2, 8) = 83.8, *p* < .001, *Ƞ* ^2^ *_p_* = 0.95). A highly significant interaction between *time‐of‐day* and *day* condition emerged, *F*(4, 16) = 16.75, *p* < .001, *Ƞ* ^2^ *_p_* = 0.81.

To interpret these interactions, we again used a simple effects analysis, examining how each *day* condition and *cycle* number influenced the movement levels at each of the three *time‐of‐day* levels. The full ANOVA output table is presented in Table 6. The most meaningful simple effects level is the *pre‐light* interval. During this interval, regardless of *day* condition, the plants were always in the dark and had been so for at least 5 h. Mean levels of *pre‐light* motion were again higher for the *Light1* and *Light2* days than for *Dark* days (*Light1 mean* = 0.83, *SEM* = 0.03; *Light2 mean* = 0.86, *SEM* = 0.02; *Dark mean* = 0.78, *SEM* = 0.02). The plants exhibited this anticipatory movement from the very first 60‐h cycle, adjusting to the change in day length immediately.

**Table 6:** Study Phase 1: 20‐h day duration, pre‐light time‐of‐day simple effects repeated measures anova tests of within‐subjects effects tests of within‐subjects effects

Tests of Within‐Subjects Effects						
Source	Sum of Squares	df	*Mean* Square	*F*	*p*	*η* ^2^_*p*_
RM Factor 1	0.00352	2	0.00176	0.98	.415	0.197
RM Factor 1 × cycle	0.00465	2	0.00233	1.30	.324	0.245
Residual	0.01432	8	0.00179			

Tests of Between‐Subjects Effects						
Source	Sum of Squares	df	*Mean* Square	*F*	*p*	*η* ^2^_*p*_
Cycle	0.00601	1	0.00601	1.37	.307	0.255
Residual	0.01759	4	0.00440			

*Note*. Type III sums of squares were used for all effects.

We again calculated the difference in motion difference for the *Light1* and *Light2* days and *Dark* days as

$$\left(Light1+Light2\right)/2-Dark.$$

Fig. 4 shows the difference in observed motion during the *pre‐light* phase prior to lighted days and dark days as a function of *cycle* number. (Note that this phase of the study lasted for only 18 days, and thus there are only six 3‐day cycles for study Phase 2 in Fig. 4.) A logarithmic accommodation pattern emerged again, this time with an *r* ^2^ value of 0.47.

MotionDifference=0.05∗log(cyclenumber)+0.01

During the *light* time‐of‐day, significantly more motion was apparent in the *Light1* and *Light2* conditions than in the *Dark* condition, *F*(2,8) = 79.5, *p* < .001, *Ƞ* ^2^ *_p_* = 0.952 (*Light1 mean* = 1.40, *SEM* = 0.04; *Light2 mean* = 1.00, *SEM* = 0.03; *Dark mean* = 0.82, *SEM* = 0.02). During the *post‐light* time of the day, no significant differences or interactions emerged (*Light1 mean* = 0.82, SEM = 0.01; *Light2 mean* = 0.89, *SEM* = 0.02; *Dark mean* = 0.85, *SEM* = 0.01).

The plants seemed to rapidly shift their nyctinastic movements in accord with a *Light1‐Light2‐Dark* sequence that followed a 20‐h day. Over the course of the six 60‐h *cycles*, the time‐of‐day, according to a clock, varied tremendously. If the plants were relying on temporal information alone, the nyctinastic movement patterns should have varied tremendously as well. The plants, however, continued to exhibit movement patterns that accorded with the *Light1‐Light2‐Dark* pattern. This suggests that they were influenced by the number of lighting events and that the plants are able to keep track of enumeration information.

The plants were affected by the shift to a 20‐h day length, but it seems clear that they did not return to the starting state observed at the beginning of study Phase 1. On the very first cycle, the plants moved less during the *pre‐light* time of the first *Dark* day than for the preceding *Light* days. By the third cycle of Phase 2, the plants were producing *pre‐light* movement differences that were approximately the same as those seen near the end of study phase 1 (Fig. 4).

The plants adapted so quickly to this shift from 72‐ to 60‐h cycles that we elected to present them with something more challenging. Rather than shifting the day length once, we began shifting the day length randomly at the end of every *Light1‐Light2‐Dark* cycle. In this context, temporal information alone is a very unreliable indicator of when the lights will turn on or remain off. Tracking the number of lighting events that have been presented, however, does indicate when a period of extended darkness has begun. We explored this in Phase 3 of the study.

## 11 Phase 3: Random duration light–light–dark cycles

In Phase 2 of the study, the plant movement levels were affected by the shift in timing, but that does not imply that they are not also engaging in enumeration‐based information encoding. Enumeration‐based encoding requires an organism to abstract patterns based on directly observable stimuli. Many animals seem to encode these directly observable stimulus characteristics and use them also. Enumeration‐based encoding seems to emerge as a “last resort,” when simpler encoding fails to predict future events (Brannon & Terrace, 1998; Davis & Pérusse, 1988).

We certainly do not seek to argue that plants are not influenced by temporal information. Like other studies of enumeration‐based processing, we seek to place *M. pudica* into a situation in which only enumeration‐based processing will be effective. In Phase 3 of the study, we randomly changed the day length at the end of every *Light1‐Light2‐Dark* cycle. Under these circumstances, the plants cannot learn the length of the lighting cycle over the course of many repetitions. Time is no longer an effective predictor of when the lights will turn on and off in this context. Enumeration remains a perfect predictor. After two *Light* day events, one can predict that an extended period of *Darkness* will follow.

### 11.1 Method

The same plants used in Phases 1 and 2 of the study continued into Phase 3. We used the same tent, lighting, image collection, and watering protocols. The only thing we changed was the timing of the lighting cycle. On the first day of Phase 3, we randomly selected a day length between 10 and 32 h. We again followed the *Light1‐Light2‐Dark* pattern used in Phases 1 and 2 but using this new day length. For instance, if a day length of 16 h was selected, then the plants would receive dark for 4 h, then light for 8 h, and then dark for 4 h (*Light1*). This would repeat over the next 16 h (*Light2*). This would be followed by 16 h of darkness (*Dark*). In general, for any day length *N*, the pattern would be dark for *N*/4 h, light for *N*/2 h, and dark for *N*/4 h (*Light1*). This would repeat for *Light2*. This would be followed by *N* h of darkness. At the conclusion of this *Light1‐Light2‐Dark* sequence, a new random day length was selected, and the process was repeated.

### 11.2 Design and analysis

The study included a fully within‐subjects design, which we analyzed using repeated‐measures ANOVA: *Time‐of‐day* (three levels: *pre‐light*, *light‐on*, *post‐light*), *Day* condition (three levels: *Light1*, *Light2*, *Dark*), *Cycle* number (30 levels: cycles 1–30), *Day Length* (11 levels: 10–30 h). For *Day* and *Time‐of‐day* repeated‐measures factors, the numerator degrees of freedom correspond to the number of levels minus one (*Day*, 2 df; *Time‐of‐day* 2 df). *Cycle* number and *Day Length* were treated as covariates. For *Cycle*, this resulted in 30 − 2 = 28 df. For *Day Length*, this resulted in 11 − 2 = 9 df.

### 11.3 Results and discussion

We again used repeated measures ANOVA to explore motion levels at each of the three *times‐of‐day* (*pre‐light, light‐on, post‐light*), with each of the 3 *day* conditions (*Light1, Light2, Dark*), across the 30 3‐day *cycles* for which we collected usable data. We entered *day length* into the model as a covariate. Group descriptive statistics and the full ANOVA output table are presented in Tables 7 and 9.

**Table 7:** Study Phase 3, variable‐hour day duration: Descriptive statistics for motion levels

Day Condition/Time‐of‐Day	*N*	*Mean*	*SEM*
L1 Pre‐light	30	1.24	0.018
L2 Pre‐light	30	1.28	0.021
D1 Pre‐light	30	1.24	0.019
L1 Light‐on	30	1.07	0.043
L2 Light‐on	30	1.05	0.050
D1 Light‐on	30	1.22	0.019
L1 Post‐light	30	1.29	0.023
L2 Post‐light	30	1.24	0.020
D1 Post‐light	30	1.25	0.015

A main effect of *day* condition emerged, *F*(2,54) = 7.37, *p* = .01, *Ƞ* ^2^ *_p_* = 0.21 (*Light1 mean* = 1.20, *SEM* = 0.03; *Light2 mean* = 1.19, *SEM* = 0.03; *Dark mean* = 1.24, *SEM* = 0.02). Significant two‐way interactions were apparent between *day* condition and *cycle* number (*F*(2, 54) = 4.4, *p* = .02, *Ƞ* ^2^ *_p_* = 0.14), between *time‐of‐day* and *cycle* number (*F*(2, 54) = 13.0, *p* < .001, *Ƞ* ^2^ *_p_* = 0.33), and between *time‐of‐day* and *day* condition (*F*(4, 108) = 7.16, *p* < .001, *Ƞ* ^2^ *_p_* = 0.21). A significant three‐way interaction was present between *day, time‐of‐day*, and *cycle* number, *F*(4, 108) = 7.2, *p* < .001, *Ƞ* ^2^ *_p_* = 0.21. A second significant three‐way interaction was present between *day, time‐of‐day*, and *day length*, *F*(4, 108) = 3.44, *p* = .01, *Ƞ* ^2^ *_p_* = 0.11.

These interactions again suggest that the plants’ response to lighting events changed substantially over the course of the study and interacted meaningfully with the length of the day. The most relevant effect identified in Phases 1 and 2 of the study was the difference between *pre‐light* movements of the plants on *Light* and *Dark* days. Specifically, the plants consistently moved more when light could be anticipated (*Light1* and *Light2* days) than when dark could be anticipated (*Dark* days). Recall that during this *pre‐light* interval, regardless of *day* condition, the plants were always in the dark and had been so for several hours prior to the start of the interval.

**Fig. 6:**
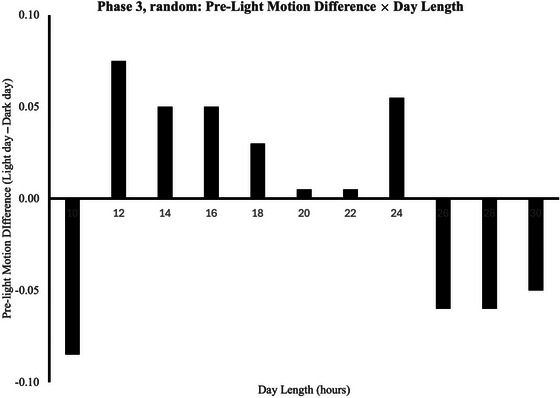
Motion differences were calculated for the *pre‐light* time of day as (*Light1* + *Light2*)/2 ‐ *Dark*. For the short, 10‐h days and days longer than 24 h, the plants moved more during the *pre‐light Dark* days than during the *pre‐light Light* days. For days between 12 and 24 h in length, the plants moved more on days when light could be anticipated than on dark days, as they did in the first two phases of the study (24‐ and 20‐h days).

Fig. 6 shows this motion difference during the pre‐light time interval as a function of the length of the day. When the randomly selected day length was between 12 and 24 h, the plants moved as they did in Phases 1 and 2 of the study—the plants moved more on *Light1* and *Light2* days and less on *Dark* days. When the day length was shorter than 12 h or longer than 24 h, the plants exhibited the opposite tendency. Group descriptive statistics for *day lengths* between 12 and 24 h are presented in Table 8.

**Table 8:** Study Phase 3, variable‐hour day durations between 12 and 24 h: Descriptive statistics for motion levels

Day Condition/Time‐of‐Day	*N*	*Mean*	*SEM*
L1 Pre‐light	23	1.257	0.021
L2 Pre‐light	23	1.301	0.021
D1 Pre‐light	23	1.241	0.019
L1 Light‐on	23	1.037	0.045
L2 Light‐on	23	1.000	0.059
D1 Light‐on	23	1.237	0.018
L1 Post‐light	23	1.283	0.021
L2 Post‐light	23	1.262	0.019
D1 Post‐light	23	1.248	0.018

**Table 9:** Study Phase 3: Variable‐hour day duration, repeated measures anova tests of within‐subjects effects

Tests of Within‐Subjects Effects						
Source	Sum of Squares	df	*Mean* Square	*F*	*p*	*η* ^2^_*p*_
Time‐of‐day	0.0837	2	0.04183	1.93	.155	0.067
Time‐of‐day × cycle	0.5616	2	0.28081	12.97	< .001	0.325
Time‐of‐day × DayLength	0.0917	2	0.04583	2.12	.130	0.073
Residual	1.1688	54	0.02164			
Day condition	0.1328	2	0.06640	7.37	.001	0.214
Day condition × cycle	0.0793	2	0.03966	4.40	.017	0.140
Day condition × DayLength	0.2235	2	0.11176	12.41	< .001	0.315
Residual	0.4864	54	0.00901			
Time‐of‐day × day condition	0.1101	4	0.02753	2.40	.054	0.082
Time‐of‐day × day condition × cycle	0.3280	4	0.08200	7.16	< .001	0.210
Time‐of‐day × day condition × Daylength	0.1574	4	0.03934	3.44	.011	0.113
Residual	1.2366	108	0.01145			

Tests of Between‐Subjects Effects						
Source	Sum of Squares	df	*Mean* Square	*F*	*p*	*η* ^2^_*p*_
Cycle	0.0406	1	0.0406	0.72	.404	0.026
Day length	0.2047	1	0.2047	3.62	.068	0.118
Residual	1.5249	27	0.0565			

To further explore the dynamics of this process, we calculated the motion difference again as

$$\left(Light1+Light2\right)/2-Dark.$$

For day lengths between 12 and 24 h, the pattern from Phases 1 and 2 was present. The data were approximated by the logarithmic function (Fig. 4).

MotionDifference=0.01∗log(cyclenumber)+0.01.

For the shortest day length (10 h) and the day lengths greater than 24 h (26–32 h), this trend was not present. Why did the plants fail to track enumeration outside of the 12–24‐h day lengths? We speculate that there is a minimum stimulus length for the plants to succeed in enumeration‐based encoding and a maximum duration across which this encoding can be maintained.

For enumeration processing to take place, the events must be presented within an appropriate time window. If visual stimuli are presented to humans for less than about 34 ms, they cannot accurately estimate the number of stimuli that have been presented (Reeves, 1996). We speculate that there must be some minimum stimulus time for the plants to encode them. Our data suggest that this minimum time is the 6 h of light associated with the 12‐h day length condition.

When the day length was longer than 24 h, the plants also did not produce the motion difference pattern observed in prior phases of the study. In order to enumerate the events, the plants need to store information collected in the past. If humans or other animals are presented with information across a long enough time window, they will forget them. We speculate that there must be some maximum amount of time that the plants can store enumeration information. Our data suggest that the maximum interval is the 72 h associated with three 24‐h days.

Within the 12‐ to 24‐h window, the logarithmic, learning‐like function observed in prior phases of the study was apparent. Data in the random‐hour phase of the study were more variable than those of the prior studies, but the overall motion patterns of the plants suggest that they continued to enumerate the lighting exposure events, even when the duration of those events rapidly varied by large amounts.

## 12 General discussion

In the study reported here, we recorded the movement patterns of *M. pudica* plants under controlled cycles of light and darkness. We presented the plants with 2 days in which the lights were turned on, followed by a third day in which the lights were not. The plant movements in the hours prior to the onset of these illumination events changed as a function of this pattern. After two extended illumination events, the plants entered a period of reduced movement that continued through the day of darkness. On days when light was anticipated, the plants moved more in the hours prior to the onset of that light.

### 12.1 Could accumulation of fatigue or stimulation explain these results?

We had some concern that this result might somehow have been built in by the procedures used in the study. Perhaps the plants were more fatigued (or more stimulated) after 2 days of illumination than after 1 or 0 days of illumination. This could have caused the smaller levels of motion for reasons not having to do with the number of lighting events presented. To assess whether or not the plants were actually learning anything during the procedure, we looked at the emergence of the pattern observed between 12:00 and 6:00 a.m. During the first 12 days of the procedure, the lower level of pre‐*Dark* day motion was not present. Indeed, the mean difference trended in the opposite direction. The logarithmic pattern of change observed in the plants suggests a process of change that, if observed in animals, would likely be referred to as learning.

To test the extent of this learning, we shortened the days from 24 to 20 h in Phase 2 of the study. The plants immediately adapted to this. In phase 3, we randomly varied the day length every three days. The movement patterns of the plants were more variable, but within a broad range of day lengths, between 12 and 24 h, the plants continued to exhibit lower levels of motion on days when no immediate illumination would be anticipated.

We know of no mechanism of fatigue or stimulation that would logarithmically increase over the course 15 days, when the plants started exhibiting this trend, and then abruptly generalize to another day length. We know of no mechanism that would perform similarly in a random‐hour lighting condition like that of Phase 3 of this study. If such a mechanism were discovered, it could provide an alternative explanation that does not involve enumeration‐based processing.

### 12.2 Do plants exhibit reaction time and forgetting?

The analysis of the results of Phase 3 was post hoc in nature. We did not anticipate that the plants would exhibit the *pre‐light* movement differences only with day lengths between 12 and 24 h. Presuming that these effects replicate in a future study in which we predict the 12‐ and 24‐h boundary conditions before performing the study, we suggest that these values reflect characteristics of the *M. pudica* information processing system.

Studies of enumeration in humans and nonhuman animals have typically designed stimuli and presentation events such that an adult human can easily perceive and reason about them. If we repeated any of the enumeration experiments described in this paper but chose to present the stimuli 10 times as quickly, we expect that many of those results would change. If stimuli are presented too quickly, they will not be effectively processed by the study participants. This almost certainly applies to plants as well as animals. If stimuli are presented too quickly, the plants will fail to fully encode them, even if a human can. In general, plants move and respond much more slowly than animals, a tendency that leads many people to think of plants as inanimate objects (Calvo & Lawrence, 2022). Our study relied heavily on time‐lapse video to see any of the movement patterns described here.

The data from Phase 3 of our study suggest that the plant reaction time limit for illumination events is quite long. During our 10‐h day condition, the illumination events lasted for 5 h. For the purposes of enumeration‐based processing, this seems too fast. In order for the pre‐*Dark* motion reduction to take place, a minimum display duration of 6 h, associated with a 12‐h day length, seems required.

At the other temporal extreme, the plants did not exhibit the pre‐*Dark* motion reduction when the day length was greater than 24 h. We speculate that this may be associated with memory limitations for the purposes of enumeration‐based processing. If we repeated any of the animal‐based enumeration experiments described in this paper but chose to present the stimuli 100 times longer, we expect that many of those results would change. In order to succeed in enumeration‐based processing in our study, the plants needed to track the number of illumination events for 3 “days.” For a 24‐h day, this adds up to 72 h. For a 32‐h day, this adds up to 96 h. We speculate that the 72‐h duration represents the memory duration limit for this process.

### 12.3 Limitations and further directions

We have reported a three‐phase investigation examining whether *M. pudica* plants are capable of encoding and distinguishing discrete light events. The findings presented here are consistent with the hypothesis that *M. pudica* can register whether it has experienced one or two light onsets within the preceding 3‐day period. Specifically, we observed a reliable pattern of nyctinastic movement that varied systematically across experimental conditions. Under the temporal conditions used in this study, plants exhibited greater movement during the pre‐light hours when light onset was expected, consistent with the pattern of prior light exposures. These results suggest a form of experience‐dependent modulation of plant movement that may reflect rudimentary encoding of temporal or quantitative regularities.

That said, these data must be interpreted cautiously. As with any experiment, it remains uncertain how broadly these findings will generalize beyond the particular environmental and procedural context of the present study. Our analyses were conducted at the group level, combining movement data across plants. This approach is not without precedent in cognitive science, where detailed analyses of individual brain‐injured patients have provided foundational insights into human cognition and neural organization. Similarly, intensive studies of small groups can yield valuable initial evidence of complex information‐processing capacities. Nonetheless, replication across multiple, independently analyzed plants—and ideally across laboratories—will be essential to establish the robustness and generality of these effects.

It is possible that the observed movement differences reflect the collective behavior of many plants, or alternatively, that a small subset of individuals—perhaps even a single plant—accounted for the aggregate pattern. Our analytic strategy cannot distinguish between these possibilities. Future studies designed to track individual plants longitudinally will be important for clarifying the extent of inter‐plant variability in such responses and for determining whether enumeration‐like processes are widespread or idiosyncratic within the species.

The current design does not assess anticipation of non‐light events or light‐offset timing, and therefore the findings cannot yet distinguish whether the underlying mechanism is tightly coupled to light‐specific physiological processes or reflects a more general event‐enumeration capacity.

Finally, our measure of movement was necessarily indirect, based on the assumption that changes in pixel values across successive time‐lapse images accurately index plant motion. We are confident that this measure captures overall activity levels, but it does not isolate the specific components of movement involved. Nyctinastic motion arises from multiple physiological systems controlling leaflets, petioles, and stems, each potentially contributing differently to the observed signal. More refined imaging approaches—such as three‐dimensional motion capture or multispectral monitoring—may help disentangle these components and reveal how distinct motor subsystems contribute to the observed effects.

We do not claim to have demonstrated plant enumeration conclusively. Rather, our goal has been to document an intriguing and testable regularity in *M. pudica*’s behavioral patterns—one that invites replication, refinement, and comparison across taxa. We hope that these findings stimulate further investigation into how plants encode, anticipate, and respond to structured regularities in their environments, and how such capacities may relate to broader theories of information processing in living systems.

### 12.4 Conclusion

Many studies have now suggested that plants possess the ability to process complex information (Abramson & Chicas‐Mosier, 2016; Baluška, Mancuso, Volkmann, & Barlow, 2009; Calvo Garzón & Keijzer, 2011; Calvo et al., 2020; Calvo & Friston, 2017; Calvo & Lawrence, 2022; Gagliano, Abramson, & Depczynski, 2018; Gagliano, Vyazovskiy, Borbély, Grimonprez, & Depczynski, 2016; Gagliano et al., 2014). The movements produced by plants are often slow and seemingly simple, but there is increasing evidence that the plants are able to gather, store, and use information to adaptively control those movements. If those movements were produced by animals, we would likely call them behaviors. In animals, the terms gathering, storing, and using information to adapt movement are more commonly described as perception, memory, and sensory‐guided action.

In animals, we presume that perception, memory, and action control are accomplished by neuronal tissues, and there is a great deal of evidence to support that assertion. But it may be that non‐neuronal tissues in both plants and animals are also capable of participating in these processes. At least some recent work has suggested, for instance, that kidney cells may learn and store information that modulates later processing according to principles that have been observed in other human memory tasks (Kukushkin, Carney, Tabassum, & Carew, 2024). “Extra‐neuronal” learning and action control may be a neglected aspect of cellular processing that can be studied in both plants and animals.

## Data Availability

All videos and Matlab analysis scripts are available online at https://osf.io/j9g2e/overview?view_only=f778916b8a664d06800b7cc36e80f952.
